# microRNA-Dependent Modulation of Genes Contributes to ESR1's Effect on ERα Positive Breast Cancer

**DOI:** 10.3389/fonc.2020.00753

**Published:** 2020-05-15

**Authors:** Shan Gao, Bisha Ding, Weiyang Lou

**Affiliations:** ^1^Second Clinical Medical College, Zhejiang Chinese Medical University, Hangzhou, China; ^2^Clinical Research Institute, Zhejiang Provincial People's Hospital, Hangzhou, China; ^3^Department of Breast Surgery, The First Affiliated Hospital, College of Medicine, Zhejiang University, Hangzhou, China

**Keywords:** microRNA (miRNA), ESR1, ERalpha (ERα), breast cancer, prognosis

## Abstract

**Background:** Dysregulation of ESR1 accounts for endocrine therapy resistance and metastasis of ERα positive breast cancer. However, the underlying molecular mechanism of ESR1 in ERα positive breast cancer remains insufficiency. Notably, to date, a comprehensive miRNA-mRNA regulatory network involved in modulation of ESR1 in development and progression of ERα positive breast cancer is still not established.

**Methods:** Microarray miRNA and mRNA expression profiling from GEO database were used to obtained significant DE-miRNAs and DE-mRNAs in ERα positive breast cancer. Functional enrichment analysis was conducted by Enrichr database. STRING database was utilized to construct protein-protein interaction network, after which hub genes were identified through Cytoscape. Kaplan-Meier plotter was introduced to perform survival analysis. The relationship between ESR1-miRNA or miRNA-target gene pairs were experimentally validated.

**Results:** 74 DE-miRNAs, including 19 upregulated and 55 downregulated miRNAs, and 830 DE-mRNAs, including 359 upregulated and 471 downregulated mRNAs, in ERα positive breast cancer were identified. Potential DE-mRNAs were statistically enriched in several cancer-associated pathways, such as cell cycle and pathway in cancer. Fifty-one hub genes with node degree more than 10 were screened. Twenty-seven of 51 hub genes had significant prognostic values in ERα positive breast cancer. Based on the 27 hub genes, a miRNA-hub gene network, containing 26 miRNAs, was established. Seven of 26 miRNAs were found to possess prognostic predictive roles for patients with ERα positive breast cancer by combination of TCGA and METABRIC data. Intriguingly, ESR1 positively correlated and regulated the 7 miRNAs and the 7 miRNAs inversely correlated and modulated their corresponding downstream targets in MCF-7 and T47D cells, supporting the accuracy of *in silico* analysis. The relationship between ESR1-miRNA, miRNA-mRNA, or ESR1-mRNA pairs was validated in clinical ERα positive breast cancer.

**Conclusions:** In total, the current findings from this work add substantially to the understanding of ESR1's molecular regulatory mechanism in ERα positive breast cancer.

## Background

Breast cancer is the most common type of cancer and the second prevalent cause of cancer-associated deaths in women worldwide (1). In the United States, an estimate of 252,710 women will be diagnosed with breast cancer every year, with approximately 41,070 breast cancer-associated deaths (2). As the complexity of breast cancer, there are more than 20 various histological subtypes and at least four distinct molecular subtypes (3, 4). Among these subtypes, up to 70% of all breast cancers are estrogen receptor alpha (ERα) positive and belong to the molecular subtypes, luminal A and luminal B (5). ERα, encoded by gene ESR1, is an estradiol-activated transcription factor. Numerous studies have suggested that ERα signaling is critical for growth of ERα positive breast cancer, links to favorable patients' prognosis and correlates with good performance in responding to endocrine therapy, including selective estrogen receptor modulators (tamoxifen) and selective estrogen receptor down regulators (fulvestrant) (6). Elucidating molecular action mechanism of ERα in ERα positive breast cancer not only offers theoretical foundation for future basic research in this field but also provides key clues for seeking and developing diagnostic, therapeutic and prognostic biomarkers of ERα positive breast cancer.

microRNAs (miRNAs) are a group of small, single-stranded, non-coding, endogenous RNA of ~22 nucleotides in size (7). miRNAs negatively modulate target gene expression, thereby serving as vital players in regulating a variety of biological processes, including proliferation, chemoresistance, angiogenesis, invasion, and metastasis (8–11). To date, several studies have reported that miRNAs directly or indirectly influence expression of ERα, thus involving in regulating growth, invasion, metastasis, and sensitivity of endocrine therapy in breast cancer. For example, miR-22 was found to inhibit breast cancer progression by directly targeting ESR1 (12); miR-873 enhanced tamoxifen sensitivity *via* indirect suppression of transcriptional activity of ERα by targeting *CDK3* which is key for phosphorylating ERα (13); miR-142-3p acted as a tumor suppressor in ER-positive breast cancer by targeting ESR1 (14). However, to the best of our knowledge, reports regarding miRNAs and their target genes regulated by ERα in breast cancer, especially ERα positive breast cancer, remain absent.

In this study, we successfully established a comprehensive ESR1-regulated miRNA-mRNA regulatory system in ERα positive breast cancer by combination of a series of *in silico* analyses and experimental validation. Intriguingly, every miRNA or mRNA in this constructed miRNA-mRNA regulatory system possesses significant predictive role for prognosis of patients with ERα positive breast cancer. These findings may lay a foundation of ESR1-mediated miRNA-mRNA regulatory mechanism on ERα positive breast cancer and seek promising prognostic biomarkers for these patients.

## Materials and Methods

### Microarray Expression Profiling

To establish a ESR1-mediated miRNA-mRNA regulatory network in ERα positive breast cancer, we searched for the potential datasets in the Gene Expression Omnibus database (http://www.ncbi.nlm.nih.gov/geo/). Only datasets met the following criteria were first included: (1) these data should be derived from tumor tissues of patients with ERα positive breast cancer; (2) included datasets should contain miRNA and mRNA transcriptome data. Then, the abstracts and corresponding information of the datasets of interest were further evaluated. Finally, only one dataset GSE38280, containing miRNA sub-dataset GSE38278 and mRNA sub-dataset GSE38279, was selected for subsequent analysis. Sub-dataset GSE38278, based on the platform of 3D-Gene Human miRNA V14_1.0.1 (GPL13746), contained 4 luminal A breast cancer samples (GSM937932-GSM937935) and 4 luminal B breast cancer samples (GSM937936-937939). Sub-dataset GSE38279, based on the platform of 3D-Gene Human Oligo chip 25k (GPL5639), contained 4 luminal A breast cancer samples (GSM937940-GSM937943) and 4 luminal B breast cancer samples (GSM937944-937947). The expression values of ESR1, ESR2, PGR, ERBB2, and MKI67 were extracted from GSE38279.

### Differential Expression Analysis

Differential expression analysis for GSE38278 and GSE38279 was performed as we previously described (15–17). Simply, the online tool GEO2R (http://www.ncbi.nlm.nih.gov/geo/geo2r), provided by the GEO database, was used to acquired the differentially expressed miRNAs (DE-miRNAs) and differentially expressed mRNAs (DE-mRNAs). Benjamini & Hochberg was applied to adjust *P*-values. |log_2_FC| > 1 and adjusted *P* < 0.05 were set as the cut-off criteria for identifying significant DE-miRNAs and DE-mRNAs.

### Prediction of Target Genes of DE-miRNAs

miRNet database (http://www.mirnet.ca/) is a user-friendly, integrated tool suite designed for comprehensive analysis and functional interpretation of miRNAs (18, 19). In this study, miRNet was employed to predict target genes of significant DE-miRNAs. The miRNA-target interactions were downloaded on the webpage.

### Drawing Veen Diagram

In order to obtain the candidate DE-mRNAs among all significant DE-mRNAs, we intersected the set of significant DE-mRNAs and the set of target gene of DE-miRNAs by drawing Veen diagram using VENNY 2.1 (https://bioinfogp.cnb.csic.es/tools/venny/index.html). The interested DE-mRNAs were considered as candidate DE-mRNAs and were chosen for following analysis. Moreover, VENNY 2.1 tool was also used to draw Veen diagram to acquire key miRNAs.

### Enrichr Database Analysis

GO functional annotation and KEGG pathway enrichment analysis for candidate DE-mRNAs was conducted by Enrichr database (http://amp.pharm.mssm.edu/Enrichr/), which is a comprehensive gene set enrichment analysis web server (20, 21). Three GO categories (biological process, cellular component, and molecular function) were included. The top 10 enriched GO and KEGG items were automatically displayed on the webpage and directly downloaded. *P* < 0.05 was considered as statistically significant.

### STRING Database Analysis

STRING database (http://string-db.org) was introduced to construct PPI network of candidate DE-mRNAs (22). The PPI network was automatically generated on the webpage.

### Identification of Hub Genes

The protein-protein interactions with combined score ≥ 0.4 were obtained by downloading the file “string_interaction.tsv” from STRING database. Subsequently, these pairs were imported into Cytoscape software (Version 3.6.1) to re-construct the PPI network. By calculating the degree of connectivity, the hub genes in the PPI network were identified *via* CytoHubba, which is a plugin in Cytoscape software (Version 3.6.1). A total of 51 hub genes (node degree ≥10) were selected for subsequent analysis.

### Kaplan-Meier Plotter Database Analysis

Kaplan-Meier plotter (http://kmplot.com/analysis/) is capable to access the effect of 54,000 genes on survival in 21 cancer types and its miRNA subsystems include 11,000 samples from 20 distinct cancer types (23, 24). Kaplan-Meier plotter was utilized to evaluate the prognostic roles of 51 hub genes in ERα positive breast cancer. After these genes were entered into the database, the hazard ratio (HR) with 95% confidence interval (CI) and logrank *P*-value were automatically calculated and displayed on the webpage. Similarly, survival analysis for candidate miRNAs in ERα positive breast cancer were also conducted using Kaplan-Meier plotter, containing data from TCGA and METABRIC databases. Logrank *P* < 0.05 was considered as statistically significant.

### Cell Culture and Clinical Samples

Two ER-positive breast cancer cell lines, MCF-7 and T47D, were purchased from the cell bank of Chinese Scientific Academy (Shanghai, China). MCF-7 and T47D were, respectively, cultured in Roswell Park Memorial Institute 1640 medium (Gibco) and Dulbecco's modified Eagle's medium (Gibco) supplemented with 10% fetal bovine serum (Thermo Scientific) and 100 U/ml penicillin, 100 μg/ml streptomycin under a humidified atmosphere of 5% CO_2_ at 37°C. The two cell lines have been identified and the third passage cells of MCF-7 and T47D were used to perform experiments in this study. Eighteen ERα positive breast cancer tissues were acquired from 18 patients who underwent surgery at the First Affiliated Hospital of Zhejiang University, College of Medicine (Hangzhou, China) and each written informed consent from every participant was obtained.

### Cell Transfection

MCF-7 and T47D cells were transfected with 100 nM ESR1 siRNA or 50 nM miRNA mimics and their corresponding controls for 48 h using Lipofectamine 3000 reagent (Invitrogen, Shanghai, China) according to the manufacturer's instructions. siRNA, miRNA mimics and their negative control oligonucleotides were purchased from RiboBio Co. Ltd (Guangzhou, China). The target sequences: si-ESR1: 5′-AGGCCAAATTCAGATAATCGACG-3′; si-ESR1^*^: 5′-AGGGAAGTATGGCTATGGAATCT.

### RNA Isolation and qRT-PCR

The isolation of total RNA from cells were performed using RNAiso plus Reagent (TaKaRa biotechnology, 9109, Kusatsu, Japan) according to manufacturer's protocol. Subsequently, total RNA was reversely transcribed into complementary DNA (cDNA) by the PrimeScript™ RT Reagent Kit (TaKaRa biotechnology, RR037A, Kusatsu, Japan). Then, qRT-PCR was performed in a Roche LightCycle480 II Real-Time PCR Detection System by SYBR Premix Ex Taq (TaKaRa biotechnology, RR420A, Kusatsu, Japan). The gene primers used in this study were designed and synthesized by BGI (Beijing, China) and were listed in Table 1. miRNA primers were designed and purchased RiboBio Co. Ltd (Guangzhou, China). The expression of gene or miRNA was, respectively, normalized to GAPDH or U6, and calculated by the comparative threshold method of 2^−ΔΔCT^.

**Table 1:** The sequences of primers used in this study.

**Gene symbol**	**Primer**	**Sequences**
ESR1	Forward primer	GGGAAGTATGGCTATGGAATCTG
ESR1	Reverse primer	TGGCTGGACACATATAGTCGTT
OIP5	Forward primer	TGAGAGGGCGATTGACCAAG
OIP5	Reverse primer	AGCACTGCGTGACACTGTG
CCNB2	Forward primer	CCGACGGTGTCCAGTGATTT
CCNB2	Reverse primer	TGTTGTTTTGGTGGGTTGAACT
AURKB	Forward primer	CAGAAGAGCTGCACATTTGACG
AURKB	Reverse primer	CCTTGAGCCCTAAGAGCAGATTT
CDC20	Forward primer	GACCACTCCTAGCAAACCTGG
CDC20	Reverse primer	GGGCGTCTGGCTGTTTTCA
MYBL2	Forward primer	CCGGAGCAGAGGGATAGCA
MYBL2	Reverse primer	CCGGAGCAGAGGGATAGCA
CCNE1	Forward primer	ACTCAACGTGCAAGCCTCG
CCNE1	Reverse primer	GCTCAAGAAAGTGCTGATCCC
CHAF1B	Forward primer	AGAGGCAAGAAGCTACCGGAT
CHAF1B	Reverse primer	CTGGCGTGAGAAGCAAAGA
GAPDH	Forward primer	AATGGACAACTGGTCGTGGAC
GAPDH	Reverse primer	CCCTCCAGGGGATCTGTTTG

### Statistical Analysis

Most of the statistical analysis was done by the bioinformatic tools mentioned above. GraphPad Prism 7 software was introduced to do statistical analysis for experimental data. All experiments were performed in triplicates and the results were shown as mean ± standard deviation. Differences between two groups were assessed using Student's *t*-test. Spearman correlation analysis was used to determine expression relationship between ESR1 and miRNA or miRNA and target gene. *P* < 0.05 or logrank *P* < 0.05 was considered as statistically significant.

## Results

### Screening Significant DE-miRNAs and Candidate DE-mRNAs Between Luminal a and Luminal B Breast Cancer

**Figure 1:**
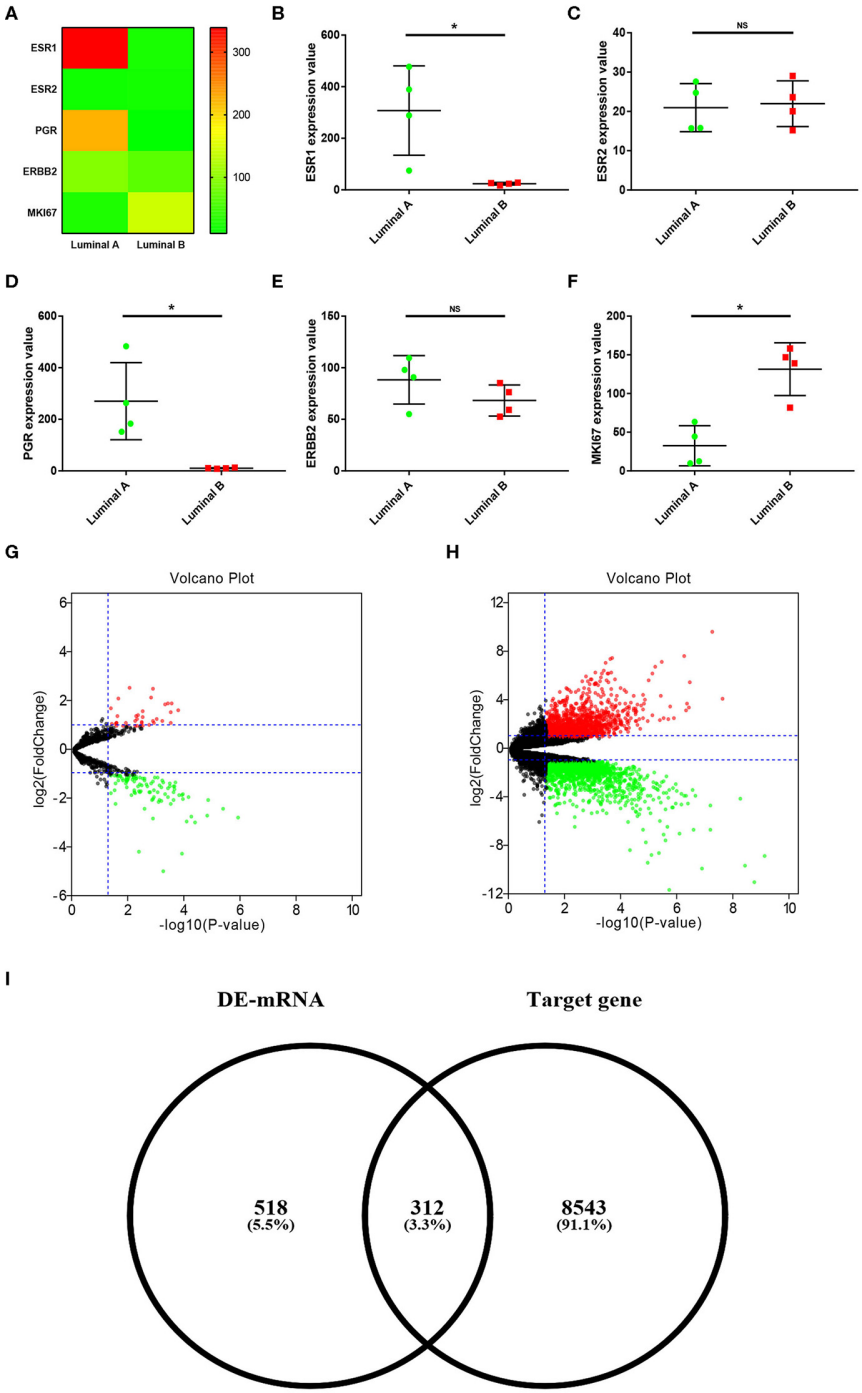
Identification of significant DE-miRNAs and candidate DE-mRNAs between luminal A and luminal B breast cancer. **(A)** The expression heatmap of several key genes (ESR1, ESR2, PGR, ERBB2, and MKI67) in ERα positive breast cancer. **(B)** ESR1 expression levels in luminal A and luminal B breast cancer. **(C)** ESR2 expression levels in luminal A and luminal B breast cancer. **(D)** PGR expression levels in luminal A and luminal B breast cancer. **(E)** ERBB2 expression levels in luminal A and luminal B breast cancer. **(F)** MKI67 expression levels in luminal A and luminal B breast cancer. The data were shown as mean ± standard deviations. “*” represented significant difference, with *P* < 0.05. **(G)** DE-miRNAs between luminal A and luminal B breast cancer. **(H)** DE-mRNAs between luminal A and luminal B breast cancer. |log_2_FC| > 1 and adjust *P* < 0.05 were set as the thresholds for identifying DE-miRNAs and DE-mRNAs. Red dots and green dots represented upregulated and downregulated DE-miRNAs (DE-mRNAs) in luminal B breast cancer compared with luminal A breast cancer, respectively. Black dots represented DE-miRNAs (DE-mRNAs) that were not differentially expressed between luminal A and luminal B breast cancer. **(I)** Identification of candidate genes by intersection of DE-mRNAs and potential target genes of DE-miRNAs.

In order to explore the ESR1-mediated miRNA-mRNA molecular regulatory mechanism in ERα positive breast cancer, dataset GSE38280 containing 4 luminal A and 4 luminal B breast cancer samples was selected in this study. First of all, we determined expression levels of ESR1 in these breast cancer samples. As shown in Figures 1A,B, ESR1 was significantly upregulated in the 4 luminal A breast cancer samples compared with that in the 4 luminal B breast cancer samples. ESR2 (encoding ERβ) expression was also assessed. As shown in Figure 1C, no significant difference between the two groups was observed. Besides, expression values of another three key molecules (PGR, ERBB2 and MKI67) in classifying molecular subtype of breast cancer were presented in Figures 1D–F, respectively. Next, miRNA sub-dataset GSE38278 of dataset GSE38280 was introduced to identify DE-miRNAs between luminal A and luminal B breast cancer using online tool GEO2R. A total of 74 significant DE-miRNAs, including 19 upregulated DE-miRNAs and 55 downregulated DE-miRNAs, were obtained as shown in Figure 1G and Supplement 1. Subsequently, we further screened DE-mRNAs between luminal A and luminal B breast cancer through performing differential expression analysis for mRNA sub-dataset GSE38279 of dataset GSE38280. As presented in Figure 1H, 359 significant upregulated DE-mRNAs and 471 significant downregulated DE-mRNAs were finally acquired. These DE-mRNAs and corresponding information of differential expression analysis were listed in Supplement 2. Then, the potential target genes of the 74 significant DE-miRNAs were predicted *via* a comprehensive database for miRNA-associated studies, namely miRNet. As presented in Supplement 3, a total of 8,855 targets were finally predicted for these DE-miRNAs. For identifying candidate DE-mRNAs, we intersected these target genes and significant DE-mRNAs. 312 DE-mRNAs were commonly appeared in the target gene set of DE-miRNAs (Figure 1I). The 312 DE-mRNAs were listed in Supplement 4 and were considered as candidate DE-mRNAs for subsequent analysis.

### Functional Annotation, Pathway Enrichment and Protein-Protein Interaction (PPI) Analysis

**Figure 2:**
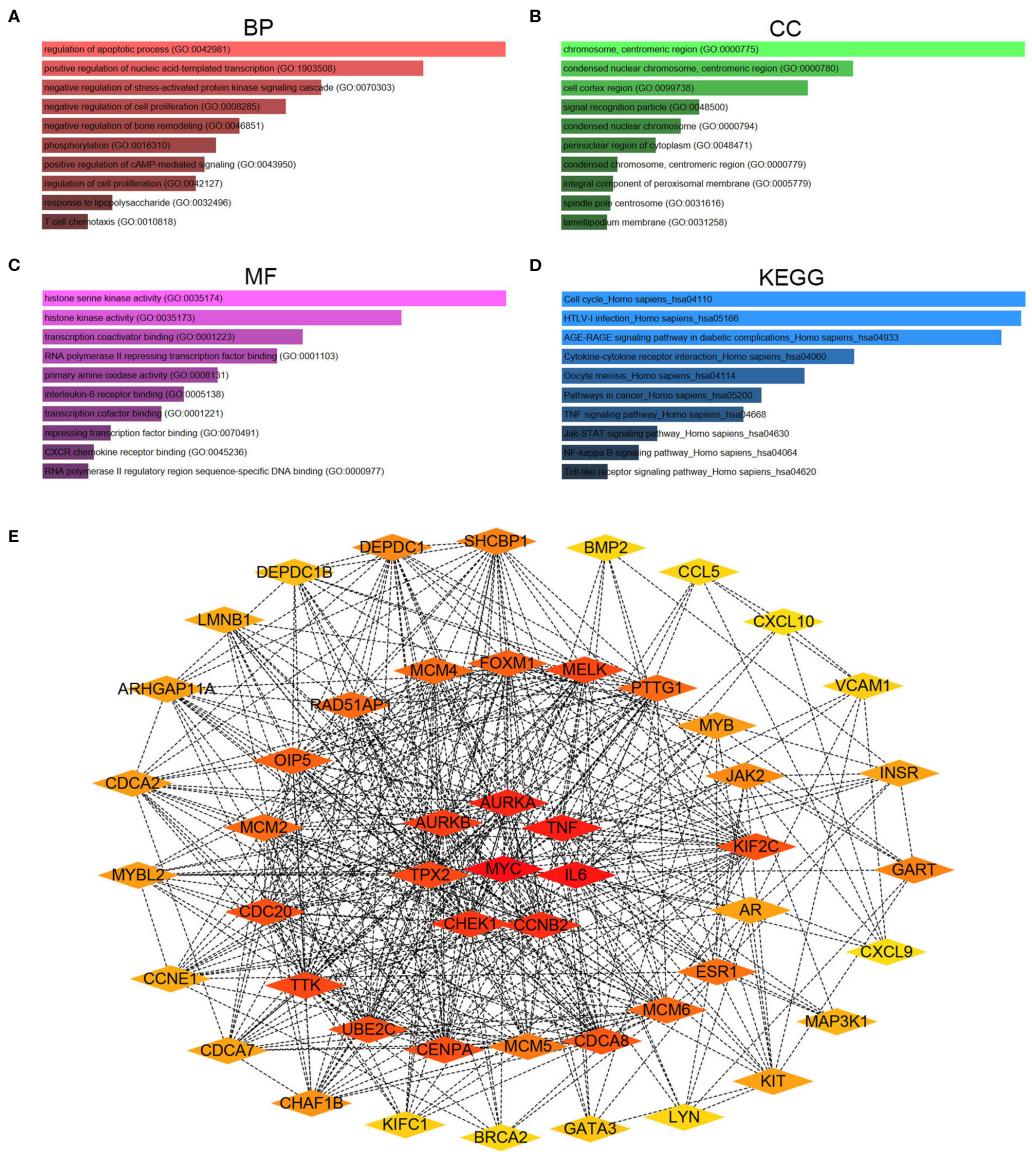
Gene Ontology (GO) functional annotation, Kyoto Encyclopedia of Genes and Genomes (KEGG) pathway enrichment and protein-protein interaction (PPI) analysis for candidate genes. **(A)** The top 10 enriched biological process (BP) items for candidate genes. **(B)** The top 10 enriched cellular component (CC) items for candidate genes. **(C)** The top 10 enriched molecular function (MF) items for candidate genes. **(D)** The top 10 enriched KEGG pathway items for candidate genes. **(E)** The PPI network of the top 51 hub genes according to node degree.

To better understand these candidate DE-mRNAs, we first performed Gene Ontology (GO) functional annotation and KEGG pathway enrichment analysis by using Enrichr database. For GO functional annotation, three categories, containing biological process (BP), cellular component (CC), and molecular function (MF), were included in this work. As shown in Figures 2A–C, the enriched GO functions for these candidate DE-mRNAs included regulation of apoptotic process, positive regulation of nucleic acid-templated transcription and negative regulation of stress-activated protein kinase signaling cascade in the BP category; chromosome, centromeric region, condensed nuclear chromosome, centromeric region, and cell cortex region in the CC category; and histone serine kinase activity, histone kinase activity and transcription coactivator binding in the MF category. For KEGG pathway enrichment analysis, we found that these candidate DE-mRNAs were significantly enriched in several cancer-associated pathways, such as cell cycle, pathway in cancer, TNF signaling pathway, Jak-STAT signaling pathway, and NF-kappa B signaling pathway (Figure 2D). Next, we mapped these candidate DE-mRNAs into STRING database for conducting PPI analysis. A variety of protein-protein interactions were observed as presented in Figure S1. Then, these interactions were re-imported into Cytoscape software (Version 3.6.1). CytoHubba was employed to identify hub genes among the PPI network according to node degree. As shown in Table 2, the top 51 hub genes with node degree ≥10 were screened. For better visualization, the sub-PPI network of the top 51 hub genes were re-constructed (Figure 2E). These hub genes were chosen for subsequent analysis.

**Table 2:** The hub genes in the protein-protein interaction (PPI) network (Degree ≥ 10).

**Gene symbol**	**Degree**
MYC	47
IL6	42
TNF	39
AURKA	37
CCNB2	35
CHEK1	34
AURKB	33
MELK	30
TTK	30
TPX2	30
KIF2C	29
UBE2C	29
CENPA	29
CDC20	29
CDCA8	28
OIP5	27
FOXM1	27
RAD51AP1	25
PTTG1	25
MCM6	25
MCM2	25
MCM4	25
ESR1	24
MCM5	23
GART	21
SHCBP1	21
DEPDC1	20
JAK2	20
CHAF1B	19
CDCA2	18
MYBL2	18
MYB	18
ARHGAP11A	17
INSR	17
CDCA7	17
KIT	17
AR	17
CCNE1	16
LMNB1	16
MAP3K1	15
DEPDC1B	14
GATA3	13
KIFC1	12
VCAM1	12
LYN	11
BRCA2	11
BMP2	11
CCL5	11
HIST3H2BB	10
CXCL9	10
CXCL10	10

### Survival Analysis for Hub Genes in ERα Positive Breast Cancer

**Figure 3:**
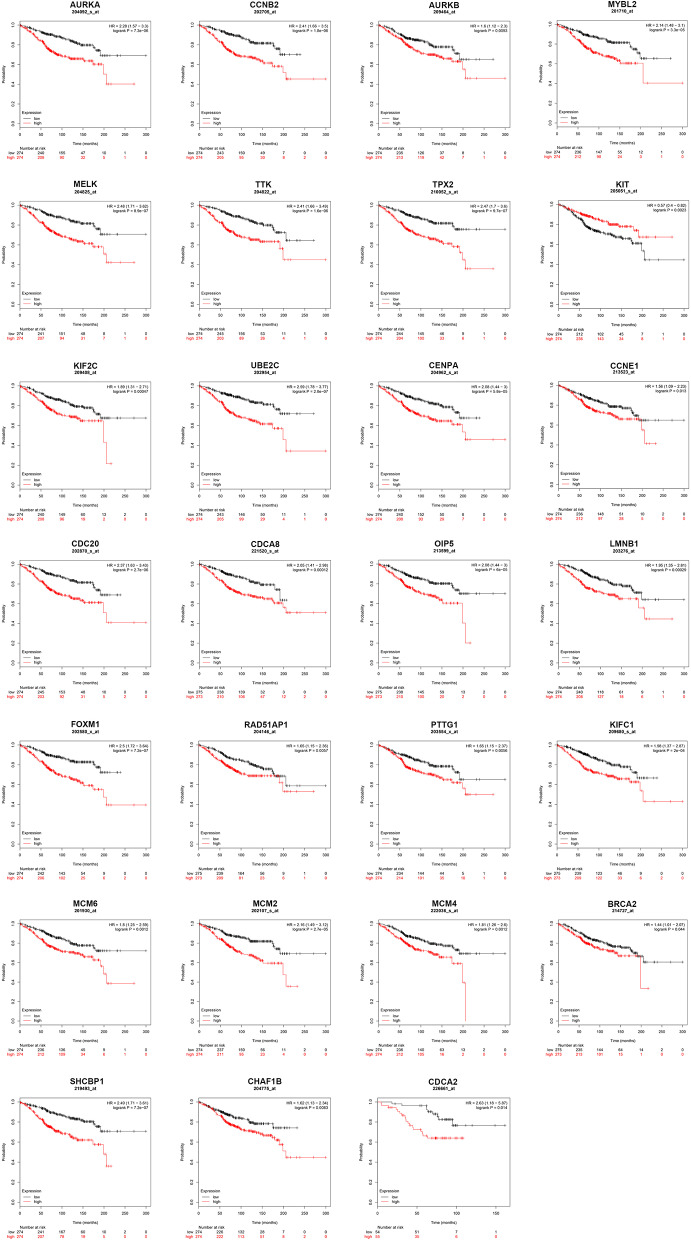
The prognostic values of key genes (*AURKA, CCNB2, AURKB, MYBL2, MELK, TTK, TPX2, KIT, KIF2C, UBE2C, CENPA, CCNE1, CDC20, CDCA8, OIP5, LMNB1, FOXM1, RAD51AP1, PTTG1, KIFC1, MCM6, MCM2, MCM4, BRCA2, SHCBP1, CHAF1B*, and *CDCA2*) in ERα positive breast cancer determined by Kaplan-Meier plotter database.

Next, we evaluated the prognostic values of the 51 hub genes in ERα positive breast cancer by using Kaplan-Meier plotter database. As shown in Figure 3, 27 of 51 hub genes possessed significant prognostic roles in ERα positive breast cancer: AURKA [logrank *P* = 7.3e-06; HR (95% CI) = 2.28 (1.57–3.30)], CCNB2 [logrank *P* = 1.8e-06; HR (95% CI) = 2.41 (1.66–3.50)], AURKB [logrank *P* = 0.0093; HR (95% CI) = 1.60 (1.12–2.30)], MYBL2 [logrank *P* = 3.3e-05; HR (95% CI) = 2.14 (1.48–3.10)], MELK [logrank *P* = 8.9e-07; HR (95% CI) = 2.48 (1.71–3.62)], TTK [logrank *P* = 1.6e-06; HR (95% CI) = 2.41 (1.66–3.49)], TPX2 [logrank *P* = 9.7e-07; HR (95% CI) = 2.47 (1.70–3.60)], KIT [logrank *P* = 0.0023; HR (95% CI) = 0.57 (0.40–0.82)], KIF2C [logrank *P* = 0.00047; HR (95% CI) = 1.89 (1.31–2.71)], UBE2C [logrank *P* = 2.8e-07; HR (95% CI) = 2.59 (1.78–3.77)], CENPA [logrank *P* = 5.8e-05; HR (95% CI) = 2.08 (1.44–3.00)], CCNE1 [logrank *P* = 0.013; HR (95% CI) = 1.56 (1.09–2.23)], CDC20 [logrank *P* = 2.7e-06; HR (95% CI) = 2.37 (1.63–3.43)], CDCA8 [logrank *P* = 0.00012; HR (95% CI) = 2.05 (1.41–2.98)], OIP5 [logrank *P* = 6.0e-06; HR (95% CI) = 2.08 (1.44–3.00)], LMNB1 [logrank *P* = 0.00029; HR (95% CI) = 1.95 (1.35–2.81)], FOXM1 [logrank *P* = 7.2e-07; HR (95% CI) = 2.50 (1.72–3.64)], RAD51AP1 [logrank *P* = 0.0057; HR (95% CI) = 1.65 (1.15–2.36)], PTTG1 [logrank *P* = 0.0056; HR (95% CI) = 1.65 (1.15–2.37)], KIFC1 [logrank *P* = 2.0e-04; HR (95% CI) = 1.98 (1.37–2.87)], MCM6 [logrank *P* = 0.0012; HR (95% CI) = 1.80 (1.25–2.59)], MCM2 [logrank *P* = 2.7e-05; HR (95% CI) = 2.16 (1.49–3.12)], MCM4 [logrank *P* = 0.0012; HR (95% CI) = 1.81 (1.26–2.60)], BRCA2 [logrank *P* = 0.044; HR (95% CI) = 1.44 (1.01–2.07)], SHCBP1 [logrank *P* = 7.2e-07; HR (95% CI) = 2.49 (1.71–3.61)], CHAF1B [logrank *P* = 0.0083; HR (95% CI) = 1.62 (1.13–2.34)] and CDCA2 [logrank *P* = 0.014; HR (95% CI) = 2.63 (1.18–5.87)]. The 27 hub genes may be key players in regulating ERα positive breast cancer.

### Identification of a Potential miRNA-mRNA Network in ERα Positive Breast Cancer

**Figure 4:**
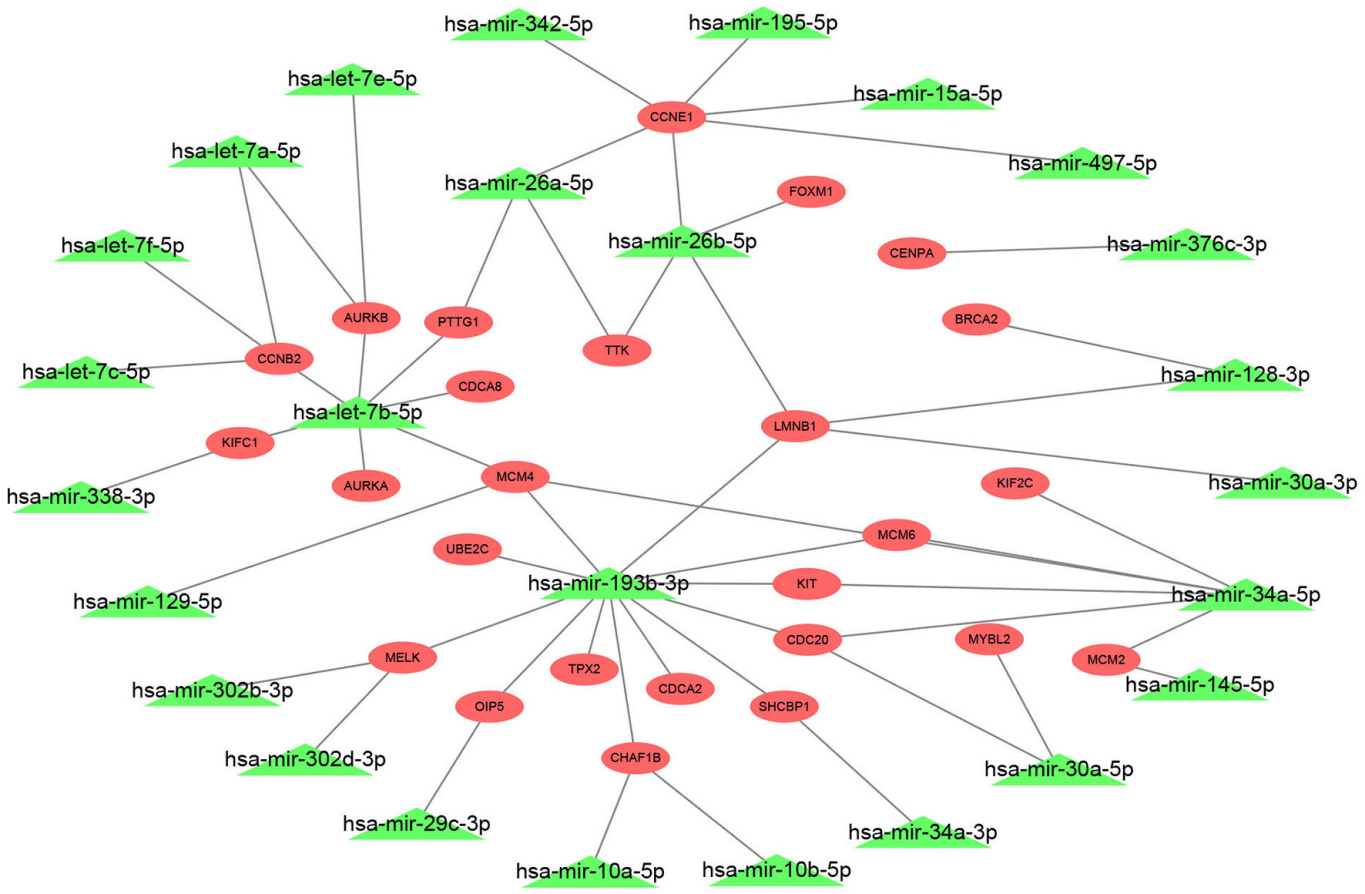
Construction of a potential miRNA-mRNA regulatory network in ERα positive breast cancer. The genes in red ellipse represented that they were significantly upregulated in luminal B breast cancer compared with luminal A breast cancer; the miRNAs in green triangle represented that they were markedly downregulated in luminal B breast cancer compared with luminal A breast cancer.

The 27 hub genes with significant prognostic values in ERα positive breast cancer were selected for constructing miRNA-mRNA network. We found the potential upstream binding miRNAs of the 27 hub genes according to Supplement 3. We also noted that the 27 hub genes were significantly upregulated in luminal B breast cancer compared with luminal A breast cancer by search of Supplement 2. Based on the negative regulatory mechanism of miRNAs on downstream target genes, the upstream miRNAs of the 27 hub genes should be downregulated in luminal B breast cancer. Among all potential miRNAs, expression levels of 26 miRNAs were significantly decreased in luminal B breast cancer. Finally, a total of 72 miRNA-mRNA pairs were found as listed in Supplement 5. For better visualization, a potential miRNA-mRNA regulatory network in ERα positive breast cancer was established as presented in Figure 4.

### Survival Analysis for Candidate miRNAs in ERα Positive Breast Cancer

**Figure 5:**
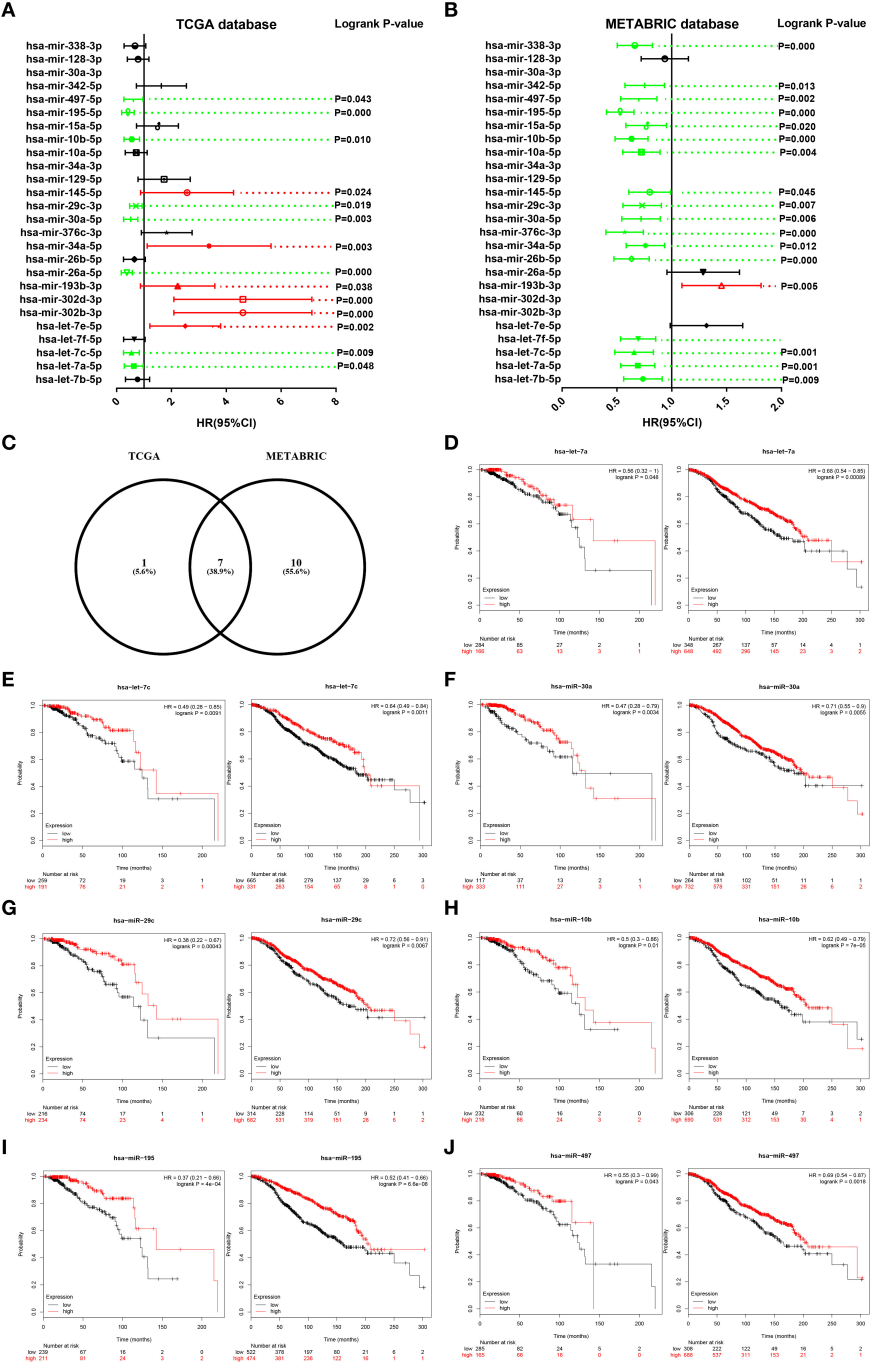
Survival analysis for candidate miRNAs in ERα positive breast cancer based on data from TCGA and METABRIC databases. **(A)** The prognostic values of candidate miRNAs in ERα positive breast cancer based on data from TCGA database. **(B)** The prognostic values of candidate miRNAs in ERα positive breast cancer based on data from METABRIC database. Green bars indicated favorable prognosis; red bars indicated unfavorable prognosis; black bars represented no statistical significance. **(C)** The intersection of miRNA with good prognosis in ERα positive breast cancer based on TCGA and METABRIC databases. **(D)** The prognostic role of hsa-let-7a-5p in TCGA (left) and METABRIC (right) ERα positive breast cancer. **(E)** The prognostic role of hsa-let-7c-5p in TCGA (left) and METABRIC (right) ERα positive breast cancer. **(F)** The prognostic role of hsa-miR-30a-5p in TCGA (left) and METABRIC (right) ERα positive breast cancer. **(G)** The prognostic role of hsa-miR-29c-3p in TCGA (left) and METABRIC (right) ERα positive breast cancer. **(H)** The prognostic role of hsa-miR-10b-5p in TCGA (left) and METABRIC (right) ERα positive breast cancer. **(I)** The prognostic role of hsa-miR-195-5p in TCGA (left) and METABRIC (right) ERα positive breast cancer. **(J)** The prognostic role of hsa-miR-497-5p in TCGA (left) and METABRIC (right) ERα positive breast cancer.

Then, we determined the prognostic roles of the 26 miRNAs in ERα positive breast cancer based on TCGA and METABRIC databases using Kaplan-Meier plotter. As shown in Figure 5A, high expression of hsa-let-7a-5p, hsa-let-7c-5p, hsa-miR-26a-5p, hsa-miR-30a-5p, hsa-miR-29c-3p, hsa-miR-10b-5p, hsa-miR-195-5p, and hsa-miR-497-5p indicated favorable prognosis whereas high expression of hsa-let-7e-5p, hsa-miR-302b-3p, hsa-miR-302d-3p, hsa-miR-193b-3p, hsa-miR-34a-5p, and hsa-miR-145-5p showed unfavorable prognosis in TCGA ERα positive breast cancer. As presented in Figure 5B, hsa-let-7b-5p, hsa-let-7a-5p, hsa-let-7c-5p, hsa-let-7f-5p, hsa-miR-26b-5p, hsa-miR-34a-5p, hsa-miR-376c-3p, hsa-miR-30a-5p, hsa-miR-29c-3p, hsa-miR-145-5p, hsa-miR-10a-5p, hsa-miR-10b-5p, hsa-miR-15a-5p, hsa-miR-195-5p, hsa-miR-497-5p, hsa-miR-342-5p, and hsa-miR-338-3p were good prognostic biomarkers for METABRIC ERα positive breast cancer. We obtained the most potential miRNAs by combination of the favorable prognostic miRNAs from TCGA and METABRIC databases. As shown in Figure 5C, 7 miRNAs with favorable prognostic roles in ERα positive breast cancer were commonly appeared in TCGA and METABRIC databases. The survival plots of hsa-let-7a-5p, hsa-let-7c-5p, hsa-miR-30a-5p, hsa-miR-29c-3p, hsa-miR-10b-5p, hsa-miR-195-5p, and hsa-miR-497-5p were presented in Figures 5D–J. The 7 miRNAs and their corresponding downstream target hub genes were selected for following analysis.

### Establishment of a ESR1-Mediated miRNA-mRNA Regulatory Network in ERα Positive Breast Cancer

**Figure 6:**
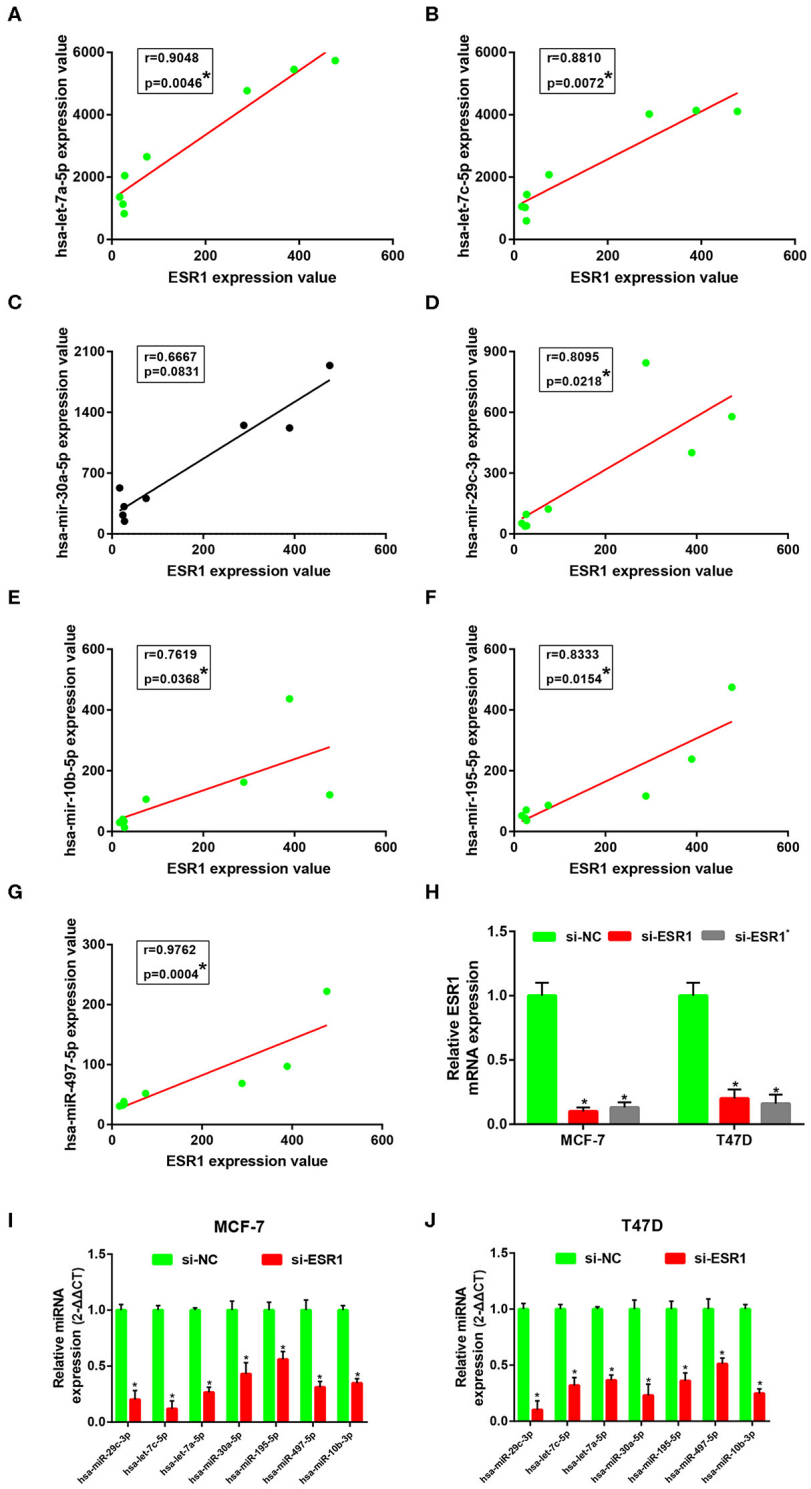
ESR1 positively regulate 7 key miRNAs in ERα positive breast cancer. **(A)** The correlation analysis of ESR1 expression and hsa-let-7a-5p expression. **(B)** The correlation analysis of ESR1 expression and hsa-let-7c-5p expression. **(C)** The correlation analysis of ESR1 expression and hsa-miR-30a-5p expression. **(D)** The correlation analysis of ESR1 expression and hsa-miR-29c-3p expression. **(E)** The correlation analysis of ESR1 expression and hsa-miR-10b-5p expression. **(F)** The correlation analysis of ESR1 expression and hsa-miR-195-5p expression. **(G)** The correlation analysis of ESR1 expression and hsa-miR-497-5p expression. “*” represented significant correlation, with *P* < 0.05. **(H)** Knockdown effect of ESR1 siRNAs in MCF-7 and T47D cell lines. **(I)** Expression change of 7 key miRNAs after silencing expression of ESR1 in MCF-7 cell line. **(J)** Expression change of 7 key miRNAs after silencing expression of ESR1 in T47D cell line. The data were shown as mean ± standard deviations. “*” represented significant difference, with *P* < 0.05.

**Figure 7:**
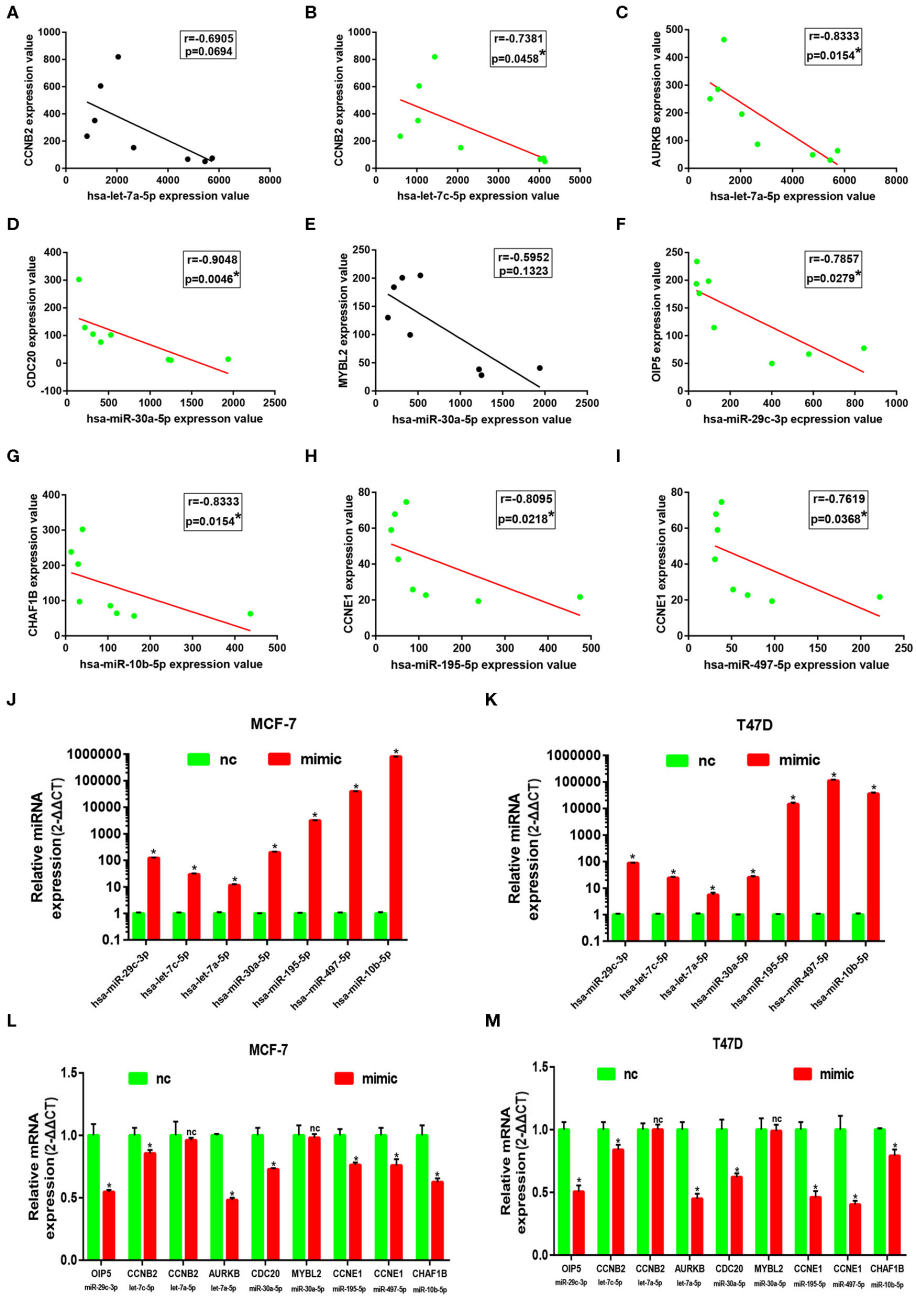
Identification of downstream genes of 7 key miRNAs in ERα positive breast cancer. **(A)** The correlation analysis of CCNB2 expression and hsa-let-7a-5p expression. **(B)** The correlation analysis of CCNB2 expression and hsa-let-7c-5p expression. **(C)** The correlation analysis of AURKB expression and hsa-let-7a-5p expression. **(D)** The correlation analysis of CDC20 expression and hsa-miR-30a-5p expression. **(E)** The correlation analysis of MYBL2 expression and hsa-miR-30a-5p expression. **(G)** The correlation analysis of CHAF1B expression and hsa-miR-10b-5p expression. **(H)** The correlation analysis of CCNE1 expression and hsa-miR-195-5p expression. **(I)** The correlation analysis of CCNE1 expression and hsa-miR-497-5p expression. “*” represented significant correlation, with *P* < 0.05. **(J)** Overexpression effect of mimics for the 7 key miRNAs at concentration of 50 nM in MCF-7 cell line. **(K)** Overexpression effect of mimics for the 7 key miRNAs at concentration of 50 nM in T47D cell line. **(L)** Expression change of downstream genes after increasing expression of corresponding miRNAs in MCF-7 cell line. **(M)** Expression change of downstream genes after increasing expression of corresponding miRNAs in T47D cell line. The data were shown as mean ± standard deviations. “*” represented significant difference, with *P* < 0.05. “nc” represented no statistical change.

**Figure 8:**
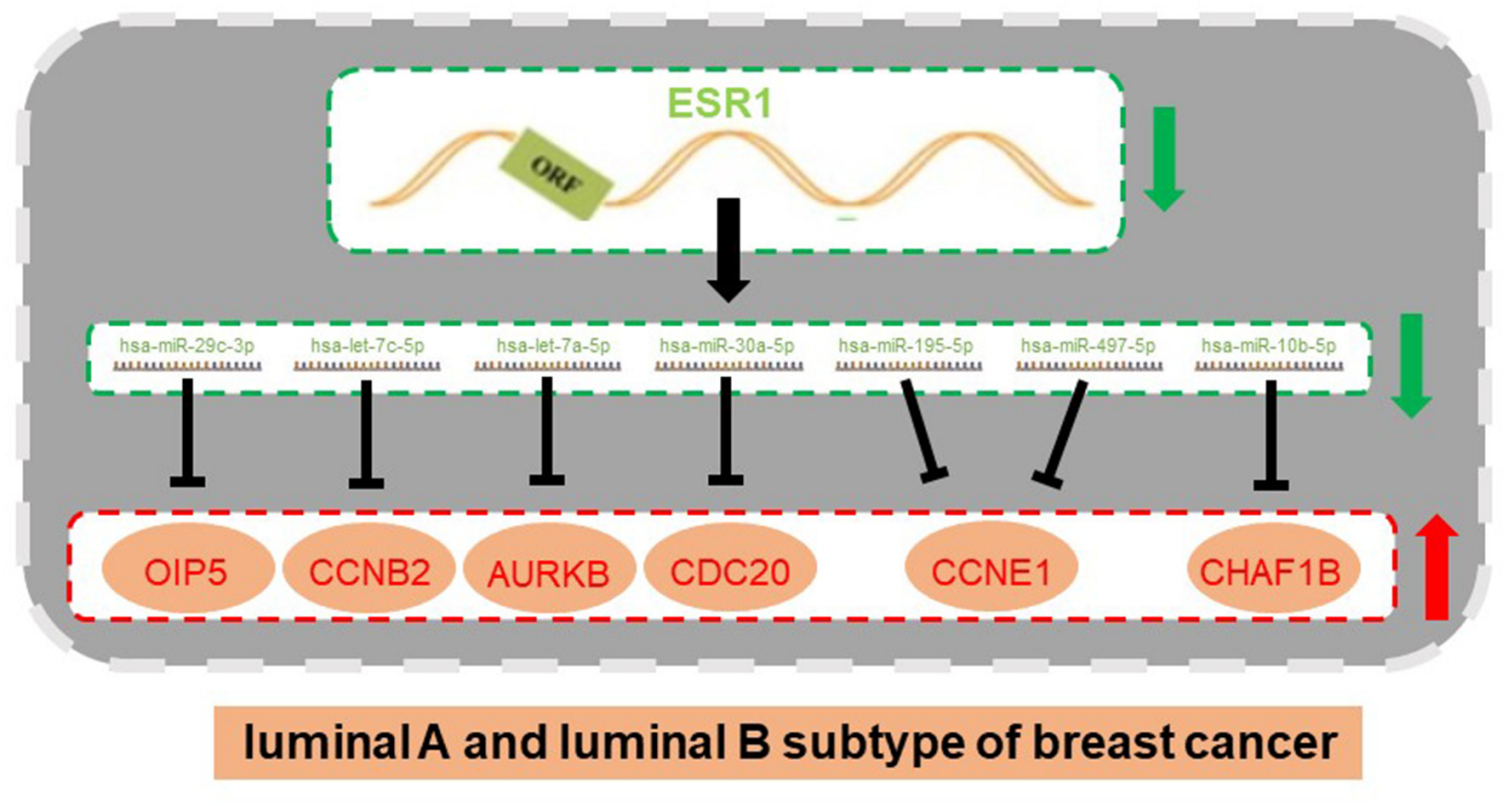
An ESR1-miRNA-mRNA regulatory network in ERα positive breast cancer (luminal B vs. luminal A breast cancer).

**Figure 9:**
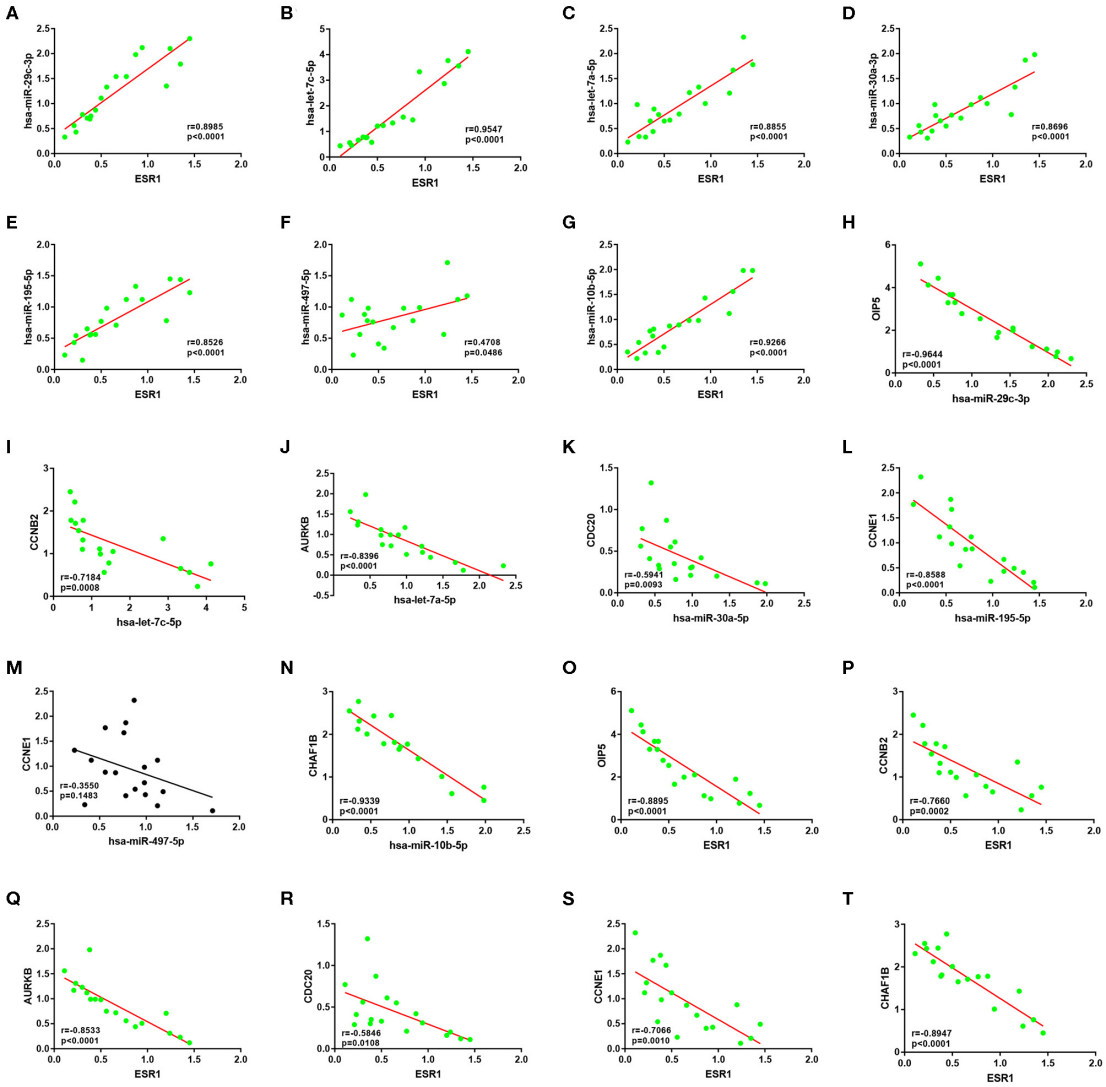
The expression correlation between ESR1-miRNA, miRNA-mRNA or ESR1-mRNA pairs of ESR1-miRNA-mRNA network in clinical ER-positive breast cancer. **(A)** ESR1/hsa-miR-29c-3p pair. **(B)** ESR1/hsa-let-7c-5p pair. **(C)** ESR1/hsa-let-7a-5p pair. **(D)** ESR1/hsa-miR-30a-3p pair. **(E)** ESR1/hsa-miR-195-5p pair. **(F)** ESR1/hsa-miR-497-5p pair. **(G)** ESR1/hsa-miR-10b-5p pair. **(H)** hsa-miR-29c-3p/OIP5 pair. **(I)** hsa-let-7c-5p/CCNB2 pair. **(J)** hsa-let-7a-5p/AURKB pair. **(K)** hsa-miR-30a-3p/CDC20 pair. **(L)** hsa-miR-195-5p/CCNE1 pair. **(M)** hsa-miR-497-5p/CCNE1 pair. **(N)** hsa-miR-10b-5p/CHAF1B pair. **(O)** ESR1/OIP5 pair. **(P)** ESR1/CCNB2 pair. **(Q)** ESR1/AURKB pair. **(R)** ESR1/CDC20 pair. **(S)** ESR1/CCNE1 pair. **(T)** ESR1/CHAF1B pair.

Subsequently, correlation analysis between ESR1 and each of the 7 miRNAs was performed. Intriguingly, ESR1 expression was positively linked to hsa-let-7a-5p (Figure 6A), hsa-let-7c-5p (Figure 6B), hsa-miR-30a-5p (Figure 6C), hsa-miR-29c-3p (Figure 6D), hsa-miR-10b-5p (Figure 6E), hsa-miR-195-5p (Figure 6F), and hsa-miR-497-5p (Figure 6G) in ERα positive breast cancer. In the next step, we wanted to ascertain if ESR1 was a potential upstream regulator of the 7 potential miRNAs. MCF-7 and T47D were chosen as two representative cell lines as high expression of ERα. ESR1 expression was significantly downregulated after using ESR1 siRNA in both MCF-7 and T47D cells (Figure 6H). Expectedly, expression levels of the 7 potential miRNAs was markedly decreased after knockdown of ESR1 in MCF-7 (Figure 6I) and T47D (Figure 6J). Next, we assessed the expression relationships of the 7 potential miRNAs and their targets in ERα positive breast cancer. As shown in Figures 7A–I, there were negative expression correlations of hsa-let-7a-5p-CCNB2, hsa-let-7c-5p-CCNB2, hsa-let-7a-5p-AURKB, hsa-miR-30a-5p-CDC20, hsa-miR-30a-5p-MYBL2, hsa-miR-29c-3p-OIP5, hsa-miR-10b-5p-CHAF1B, hsa-miR-195-5p-CCNE1, and hsa-miR-497-5p-CCNE1 in ERα positive breast cancer. Moreover, we also determined the upstream and downstream association of miRNAs and target hub genes in ERα positive breast cancer. The overexpression effect of miRNA mimics was first detected in MCF-7 and T47D cells. As shown in Figures 7J,K, the 7 miRNAs were significantly upregulated using miRNA mimics in MCF-7 and T47D, respectively. In additional to hsa-let-7a-5p-CCNB2 and hsa-miR-30a-5p-MYBL2 pairs, the expression levels of potential target hub genes of the 7 miRNAs were significantly downregulated after overexpression of the 7 miRNAs in MCF-7 (Figure 7L) and T47D (Figure 7M). Taken all these findings into consideration, an ESR1-mediated miRNA-mRNA regulatory network was constructed as shown in Figure 8. Finally, 18 ERα positive breast cancer tissues were used to validate the relationship between ESR1-miRNA, miRNA-mRNA, and ESR1-mRNA pairs (Figure 9). Intriguingly, ESR1 expression was positively correlated with miRNA expression, miRNA expression was negatively correlated with mRNA expression, and ESR1 expression was negatively correlated with mRNA expression in ERα positive breast cancer. Among these pairs, only hsa-miR-497-5p/CCNE1 pair show no statistical significance (Figure 9M), which further support our previous bioinformatic analysis.

## Discussion

More than 70% of diagnosed breast cancer cases express ERα, which is a transcription factor encoded by ESR1 located on chromosome 6 (25). It has been widely acknowledged that ERα signaling is an important event contributing to growth and disease progression of ERα positive breast cancer (26). The underlying molecular mechanism of how ESR1 acts remains unclear. miRNAs, a class small endogenous noncoding RNAs, play key roles in multiple biological processes by negatively regulating downstream gene expression (7, 11). Several miRNAs have been reported to directly target ESR1 to influence breast cancer development and progression, such as miR-22 (12), miR-301a-3p (6), and miR-206 (27). However, there are few studies about miRNAs regulated by ESR1. The purpose of the current study was to determine the ESR1-mediated miRNA-mRNA regulatory network in ERα positive breast cancer.

Based on the GEO datasets GSE38278 and GSE38279, we successively identified 74 DE-miRNAs and 830 DE-mRNAs between luminal A and luminal B breast cancer using the online tool, GEO2R. Subsequently, a widely used database miRNet was employed to predict the potential targets of DE-miRNAs. 312 candidate DE-mRNAs were commonly appeared in the target gene set of DE-miRNAs after intersection of DE-mRNAs and target genes of DE-miRNAs. GO and KEGG enrichment analyses are widely used to understand a large number of genes. Enrichment analysis revealed that they were significantly enriched in several cancer-associated pathways, such as cell cycle (28), TNF signaling pathway (29), Jak-STAT signaling pathway (30), and NF-kappa B signaling pathway (31). Next, PPI analysis to further comprehend interactive relationships among these candidate DE-mRNAs. After entering them into STRING database, a variety of protein-protein interactions were observed. To obtain hub genes among PPI network, these interactions were re-imported into Cytoscape software. Fifty-one hub genes, including AURKA, AURKB, UBE2C, and PTTG1, were screened. Most of these hub genes were reported as critical players in regulating occurrence and development of breast cancer. For example, Zhang et al. found that nuclear AURKA acquired kinase-independent transactivating function to enhance breast cancer stem cell phenotype (32); PTTG1 promoted growth, endocrine therapy resistance of breast cancer (33–35); UBE2C post-transcriptionally upregulated by miR-196a enhanced proliferation of breast cancer cell (36). Then, we performed survival analysis for the 51 hub genes. Twenty-seven of the 51 hub genes possessed significant prognostic values in ERα positive breast cancer. The 27 hub genes were upregulated in luminal B breast cancer compare with luminal A breast cancer and were selected for constructing miRNA-hub gene network.

Based on the negative action mechanism of miRNA on gene expression (37), we only included 26 miRNAs that were significantly downregulated in luminal B breast cancer compared with luminal A breast cancer. Similarly, the prognostic roles of potential miRNAs in ERα positive breast cancer were also determined using TCGA and METABRIC ERα positive breast cancer data. Finally, a total of 7 miRNAs, containing hsa-let-7a-5p, hsa-let-7c-5p, hsa-miR-30a-5p, hsa-miR-29c-3p, hsa-miR-10b-5p, hsa-miR-195-5p, and hsa-miR-497-5p, were found to be favorable prognostic biomarkers for patients with ERα positive breast cancer in both TCGA and METABRIC databases. These miRNAs have been demonstrated to functional tumor suppressive miRNAs in breast cancer. For example, Liu et al. suggested that hsa-miR-497-5p inhibited breast cancer cell proliferation, invasion, and survival by targeting SMAD7 (38); Wang et al. showed that hsa-miR-497-5p suppressed tumor growth and angiogenesis through modulating IRS1 in breast cancer (39); Li et al. found that hsa-miR-29c-3p played a suppressive role in breast cancer by inhibiting the TIMP3/STAT1/FOXO1 pathway (40). These reports together with our previous findings demonstrated that the 7 miRNAs may be important regulators in ERα positive breast cancer.

The expression relationships between ESR1 and the 7 miRNAs were subsequently evaluated. The results revealed that ESR1 were positively correlated with the 7 miRNAs in ERα positive breast cancer. Experimental validation showed that these miRNAs were significantly downregulated after knockdown of ESR1 in ERα positive breast cancer cells. Next, we also determined the expression correlation of the 7 miRNAs and their corresponding potential targets. Intriguingly, the 7 miRNAs were inversely linked to these hub genes. After overexpression of the 7 miRNAs, most of corresponding downstream target genes were downregulated in MCF-7 and T47D cells. Finally, an ESR1-mediated miRNA-mRNA network in ERα positive breast cancer were established. The relationship of pairs in this network was also validated in clinical ERα positive breast cancer. The results were in accordance with the previous analytic findings. In the future, more *in vitro* and *in vivo* functional experiments need to be performed to further confirm roles of these miRNAs and mRNAs in the established network in endocrine resistance and metastasis of ERα positive breast cancer.

## Conclusion

In conclusion, the evidence from this study suggests that a potential miRNA-mRNA network may be involved in the effect of ESR1 on ERα positive breast cancer. This study has gone some way toward enhancing our understanding of the underlying molecular mechanism of ESR1-mediated progression of ERα positive breast cancer and this research will serve as a base for future studies in this field. In the future, more basic experiments and clinical trials need to be conducted to validate these findings.

## Data Availability Statement

The datasets generated for this study can be found in the NCBI Gene Expression Omnibus (http://www.ncbi.nlm.nih.gov/geo/) (GSE38278 GSE38279 GSE38280).

## Ethics Statement

The studies involving human participants were reviewed and approved by Institutional Ethics Review Broad of the First Affiliated Hospital, College of Medicine, Zhejiang University. The patients/participants provided their written informed consent to participate in this study.

## Author Contributions

WL and SG designed this work, performed experiments, analyzed data, draft the manuscript, and polished the language. BD performed some experiments. All authors approved the final version of the manuscript.

## Conflict of Interest

The authors declare that the research was conducted in the absence of any commercial or financial relationships that could be construed as a potential conflict of interest.

## Supplementary Material

Figure S1 The protein-protein interaction (PPI) network of candidate DE-mRNAs established by STRING database.

Supplement 1 The DE-miRNAs between luminal A and luminal B breast cancer.

Supplement 2 The De-mRNAs between luminal A and luminal B breast cancer.

Supplement 3 The potential target genes of DE-miRNAs.

Supplement 4 The 312 DE-mRNAs commonly appeared in the gene set of target genes of DE-miRNAs.

Supplement 5 The 72 potential miRNA-mRNA paris in ERa positive breast cancer.
